# A serine protease KLK8 emerges as a regulator of regulators in memory: Microtubule protein dependent neuronal morphology and PKA-CREB signaling

**DOI:** 10.1038/s41598-018-27640-6

**Published:** 2018-07-02

**Authors:** Arpita Konar, Ashish Kumar, Bryan Maloney, Debomoy K. Lahiri, Mahendra K. Thakur

**Affiliations:** 1 Biochemistry and Molecular Biology Laboratory, Brain Research Centre, Department of Zoology, Banaras Hindu University, Varanasi, 221005 India; 2 CSIR-Institute of Genomics & Integrative Biology, New Delhi, 110025 India; 3 Departments of Psychiatry and of Medical and Molecular Genetics, Stark Neuroscience Research Institute, Indiana Alzheimer Disease Center, Indiana University School of Medicine, 320 West 15th Street, Indianapolis, IN 46202 USA

## Abstract

The multitude of molecular pathways underlying memory impairment in neurological disorders and aging-related disorders has been a major hurdle against therapeutic targeting. Over the years, neuronal growth promoting factors, intracellular kinases, and specific transcription factors, particularly cyclic AMP response element-binding protein (CREB), have emerged as crucial players of memory storage, and their disruption accompanies many cognitive disabilities. However, a molecular link that can influence these major players and can be a potential recovery target has been elusive. Recent reports suggest that extracellular cues at the synapses might evoke an intracellular signaling cascade and regulate memory function. Herein, we report novel function of an extracellular serine protease, kallikrein 8 (KLK8/Neuropsin) in regulating the expression of microtubule associated dendrite growth marker microtubule-associated protein (MAP2)c, dendrite architecture and protein kinase A (PKA)-CREB signaling. Both knockdown of KLK8 via siRNA transfection in mouse primary hippocampal neurons and via intra-hippocampal administration of KLK8 antisense oligonucleotides *in vivo* reduced expression of MAP2c, dendrite length, dendrite branching and spine density. The KLK8 mediated MAP2c deficiency in turn inactivated PKA and downstream transcription factor phosphorylated CREB (pCREB), leading to downregulation of memory-linked genes and consequent impaired memory consolidation. These findings revealed a protease associated novel pathway of memory impairment in which KLK8 may act as a “regulator of regulators”, suggesting its exploration as an important therapeutic target of memory disorders.

## Introduction

Memory loss is a devastating feature of neurodegenerative pathologies, traumatic brain injuries, psychiatric disorders and aging. The most common severe disorder associated with memory loss is probably Alzheimer’s disease (AD), which afflicts tens of millions, worldwide and is projected to increase indefinitely^1^. The complex molecular mechanism(s) of memory processes and multi-factorial etiology of associated disorders make recovery from AD difficult, even as a hypothesis. Neural plasticity decline is crucial for memory loss^2^, though potential therapeutic targets are elusive. Recent studies highlight extracellular synaptic proteases as emerging players of plasticity but their involvement in memory loss remains unexplored^3,4^. Amongst such proteases, Kallikrein 8 (KLK8, “neuropsin”), a serine protease^5^ of the kallikrein (KLK) family deserves more attention. KLK proteins are a large (>10 member) family of secreted serine proteases with multiple and varying expression patterns and physiological roles. KLK6, for example, is another brain-enriched kallikrein implicated in neurodegeneration, though its direct involvement in memory processes is not known. It is associated with alteration of amyloid metabolism in AD^6^, degradation of α-synuclein aggregates in Parkinson’s disease (PD) and demyelination in multiple sclerosis^7^.

KLK8 was discovered a decade ago^8^ as a neuronal activity-induced gene^9,10^ predominantly expressed in limbic regions of brain. Later, KLK8 knockout studies in rodents revealed its importance in maintaining synaptic plasticity^11,12^. Despite being such an attractive candidate, specific roles of KLK8 in memory processes and its association with memory and cognitive disorders are not yet deciphered. Herein, we report that knockdown of KLK8 in mouse hippocampus, *in vitro* and *in vivo*, impaired microtubule-associated dendrite growth and arborization, protein kinase A activation of cAMP-response element-binding protein (PKA/CREB) signaling, and object recognition memory consolidation. Microtubule-associated protein (MAP)2c has a binding domain for PKA and dendrite initiation and growth was enhanced by anchorage of PKA to this domain^13,14^. Activation of PKA pathways induces CREB phosphorylation^15–17^.

Our goal is to understand the mechanistic role of KLK8, particularly in memory. During memory formation, activity-dependent synaptic plasticity is induced at specific synapses. This step is both necessary and sufficient for the information storage that involves the type of memory specified by brain area. Furthermore, synaptic plasticity necessitates long-lasting modification of synaptic characteristics, including morphology and coupling strength. Previously, it was shown that a serine protease, KLK8, specifically alters L1 cell adhesion molecule (L1CAM), which was localized to the presynaptic site of synapse in the mouse hippocampus. Notably, increased neural activity triggered the rapid, transient activation of the precursor form of KLK8 in an NMDA receptor-dependent manner. The activated KLK8 directly cleaved L1CAM and released a KLK8-specific extracellular 180 kDa fragment^18^.

L1CAM is a cellular adhesion protein that is associated with memory establishment and maintenance, particularly repetition-assisted memory^19^. Its specific neurophysiological role is in neuronal maturation rather than proliferation, and it is upregulated mostly in later stages of neuronal differentiation^20^. In addition to facilitating neuron maturation, L1CAM binds the AD-associated amyloid-beta (Aβ) peptide and may offer direct protection against Aβ accumulation into fibrils and amyloid plaques^21^. L1CAM is subject to cleavage by several enzymes, including plasmin^18^, β-site amyloid cleaving enzyme 1 (BACE1)^22^, and KLK8^18^. Full length L1CAM is detectable on western blot as a 200–220 kDa band. Plasmin cleavage produces 120–140 and 80 kDa bands; KLK8 cleavage produces 180–200 and 20 kDa bands; and proteolytic products of both plasmin and KLK8 cleavage are 120–140, 60 and 20 kDa bands^18^. Of such fragments, only the 200–220, 80, and 20 kDa fragments would be detected by the carboxyl-terminal antibody commercially available to our laboratory.

In this study, we utilized cellular and molecular approaches to decipher the role of the secretory protease KLK8 in memory processes. Herein, we observed that KLK8 deficiency in mouse hippocampus reduced L1CAM cleavage and MAP2c levels and caused profound loss of dendrites. Further, disruption of dendrite growth impaired novel object recognition memory in KLK8 compromised mice possibly via inhibition of PKA/CREB signaling. This work is significant in unraveling the potential of a secretory protease KLK8 as an important regulator of memory markers and a probable therapeutic target.

## Results

### Hippocampal KLK8 deficiency drastically reduced microtubule associated protein (MAP)2c dependent dendrite growth

**Figure 1:**
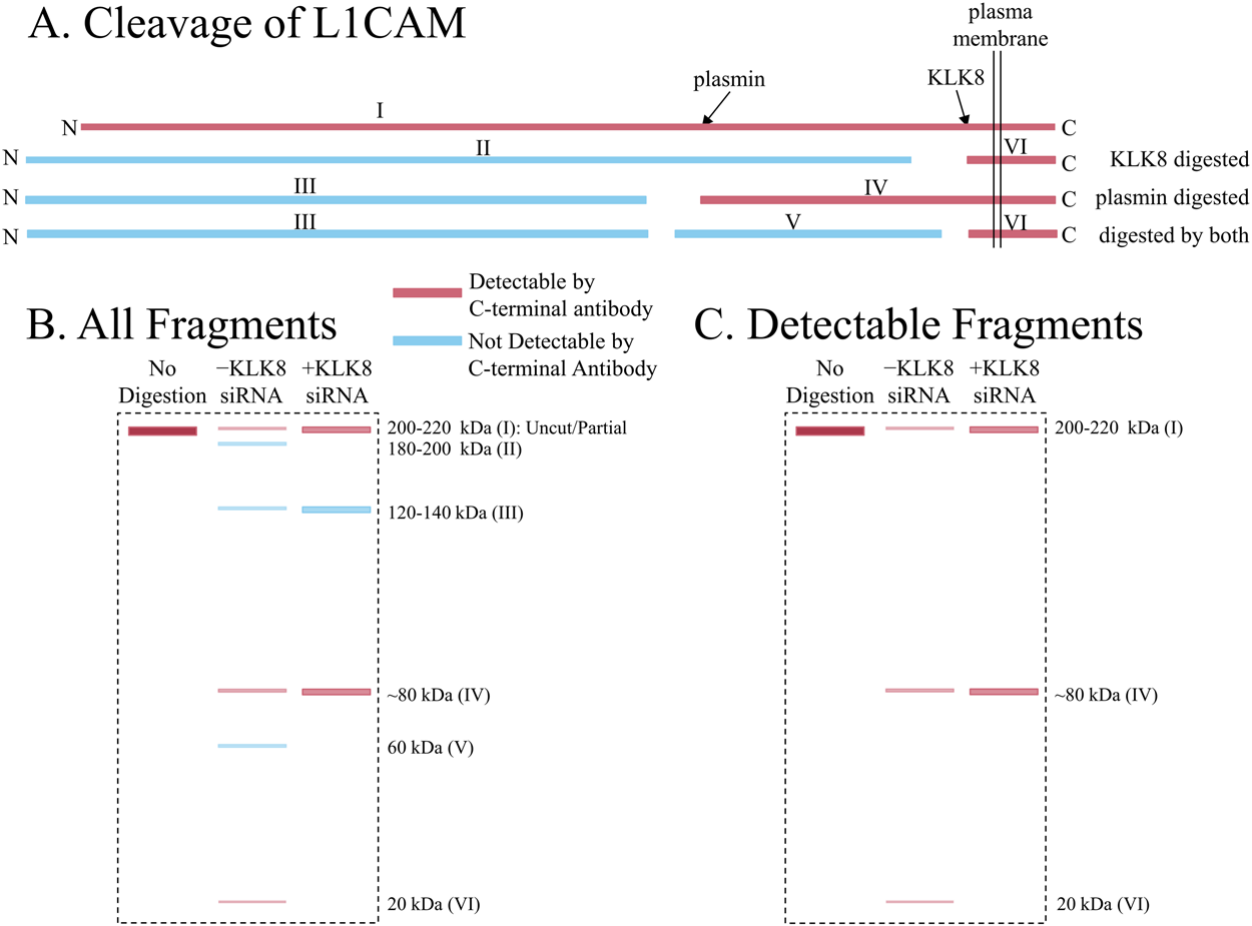
Cleavage products of L1CAM processing vs. detectability. (**A**) Cleavage products of L1CAM vs. plasmin, KLK8, or plasmin + KLK8 cleavage. Red products are detectable by a C-terminal epitope anti-L1CAM. Bands are numbered sequentially (I–VI) according to descending length from I = full-length. (**B**) Schematic of hypothetical SDS page of L1CAM cleavage. Cleavage products detectable by C-terminal epitope anti-L1CAM in red. Lane 1: Uncleaved L1CAM; lane 2: L1CAM cleaved by both plasmin and KLK8 (no siRNA interference), including partial cleavage products. Intensity and thicknesses of individual bands would be reduced from that of uncleaved L1CAM, although total intensity and area would hypothetically equal band in lane 1; lane 3: L1CAM cleavage under siRNA blockade of KLK8. Intensity of individual bands would be greater than in lane 2 and sum band density by area would hypothetically equal lane 1; lane 4. (**C**) “Detectable” (with currently-available antibodies) bands on hypothetical western blot. Lanes are as in B.

**Figure 2:**
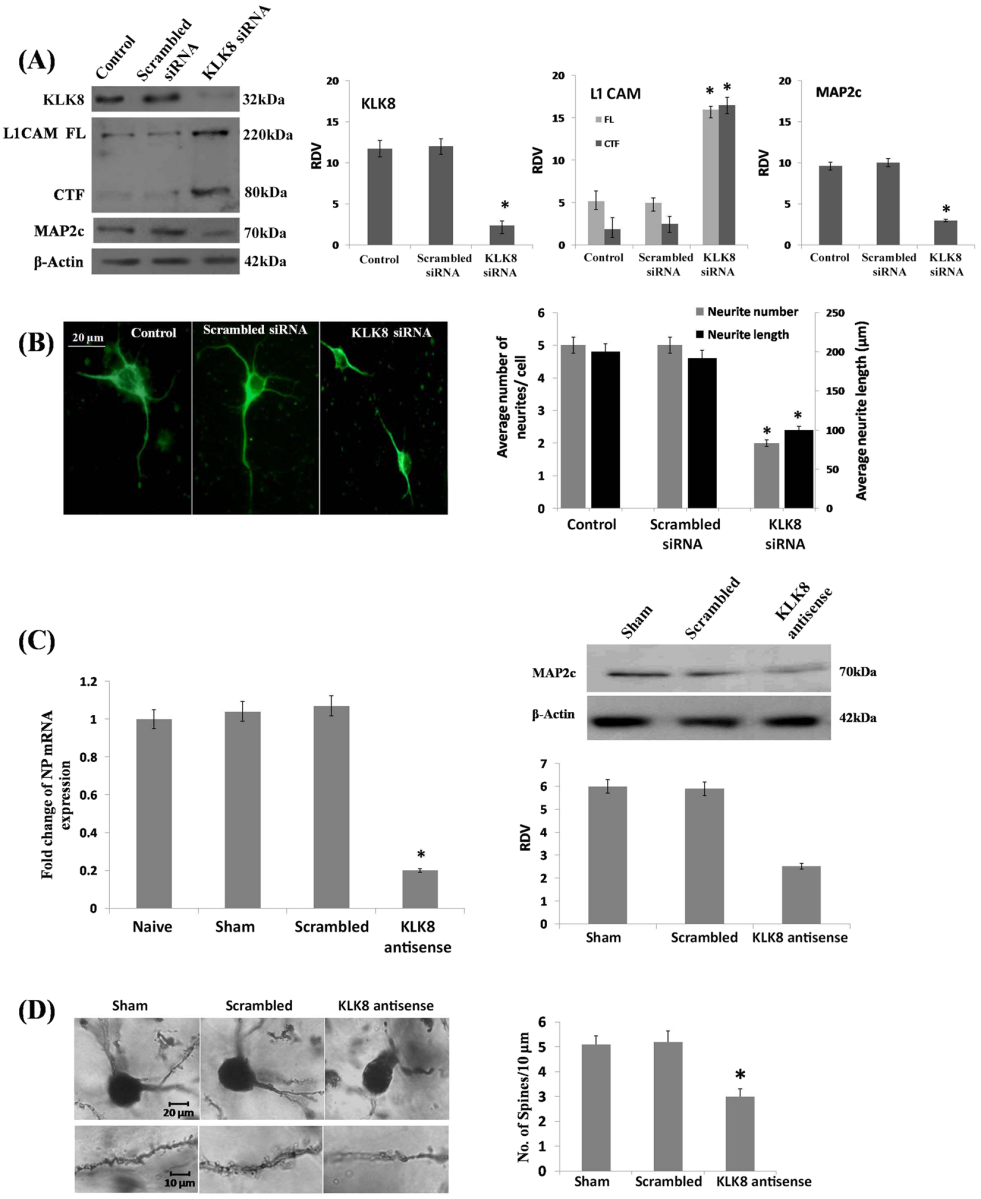
KLK8 knockdown *in* *vitro* and *in* *vivo* in mouse hippocampus markedly reduced microtubule associated dendrite growth and spine density. (**A**) Western blot analysis showing reduced L1CAM cleavage and MAP2c level and (**B**) dendrite growth in primary hippocampal neurons upon KLK8 gene silencing. ‘*’ Denotes significant differences (*p* < 0.05) as compared to control. *Silencer*® Negative siRNA with scrambled sequence was used as a negative control along with another control cells treated only with lipofectamine. (**C**) Hippocampal KLK8 mRNA expression, MAP2c level and (**D**) Golgi staining based dendrite spine density upon KLK8 knockdown by antisense oligonucleotides ‘*’ denotes significant differences as compared to sham control.

KLK8 siRNA treatment did not induce neuronal death as compared to untreated and scramble treated controls, shown by trypan blue staining based live and dead cell counting using hemocytometer, along with DAPI staining-based assessment of nuclear morphology (Fig. S1). KLK8 protein level was markedly reduced (0.80-fold) compared to lipofectamine-only and scrambled sequence treated negative control. KLK8-compromised cells showed significant decrease in L1CAM cleavage, according to increased band intensities of 200–220 (3-fold) and 80 kDa (8.68-fold) fragments. When L1CAM is cleaved at normal rates by plasmin and KLK8 combined (Fig. 1A), the process potentially results in 6 fragments (Fig. 1B), One unique double-cleavage product (60 kDa), 4 single-cleavage products (180–200, 120–140, ~80, 20 kDa), and partially uncleaved L1CAM (200–220 kDa). The only commercially-available anti-L1CAM was against a carboxyl-terminal epitope, which cannot recognize all cleavage products (Fig. 1C). Thus, cleavage products that require the activity of KLK8 were not directly detectable. However, we were able to indirectly infer results of KLK8 siRNA blockade on L1CAM cleavage. The ~80 kDa fragment (IV) is a product of plasmin activity alone. If KLK8 mediated cleavage occurs, the intensity of fragment IV would be reduced, since it would be rendered into fragments V and VI (60 and 20 kDa, respectively). In addition, levels of uncleaved L1CAM (Fragment I, 200–220 kDa) would be greatly decreased if both enzymes were active. Elimination of either would increase levels of uncleaved L1CAM (Fig. 1B,C). This is what we observed for the full-length and ~80 kDa L1CAM products vs. KLK8 suppression by siRNA (Fig. 2A). The 20 kDa product presumably ran with the salt front. MAP2c level was also reduced by 0.8-fold in KLK8-knockdown cells (Fig. 2A). Other MAP2 isoforms (MAP2a, MAP2b, and MAP2d) were unaffected (data not shown). MAP2c-stained neurons showed drastic loss in dendrite branching (0.6-fold) and length (0.5-fold) in KLK8 silenced cells (Fig. 2B). This *in*-*vitro* observation was further corroborated *in* *vivo* by infusion of KLK8 antisense oligonucleotides in mouse hippocampus. KLK8 expression knockdown by 0.8-fold drastically reduced MAP2c levels (0.58-fold, Fig. 2C), dendrite length and number of spines (Fig. 2D).

### Disruption of dendrite growth impaired novel object recognition memory in KLK8 compromised mice possibly via inhibition of PKA/CREB signaling

**Figure 3:**
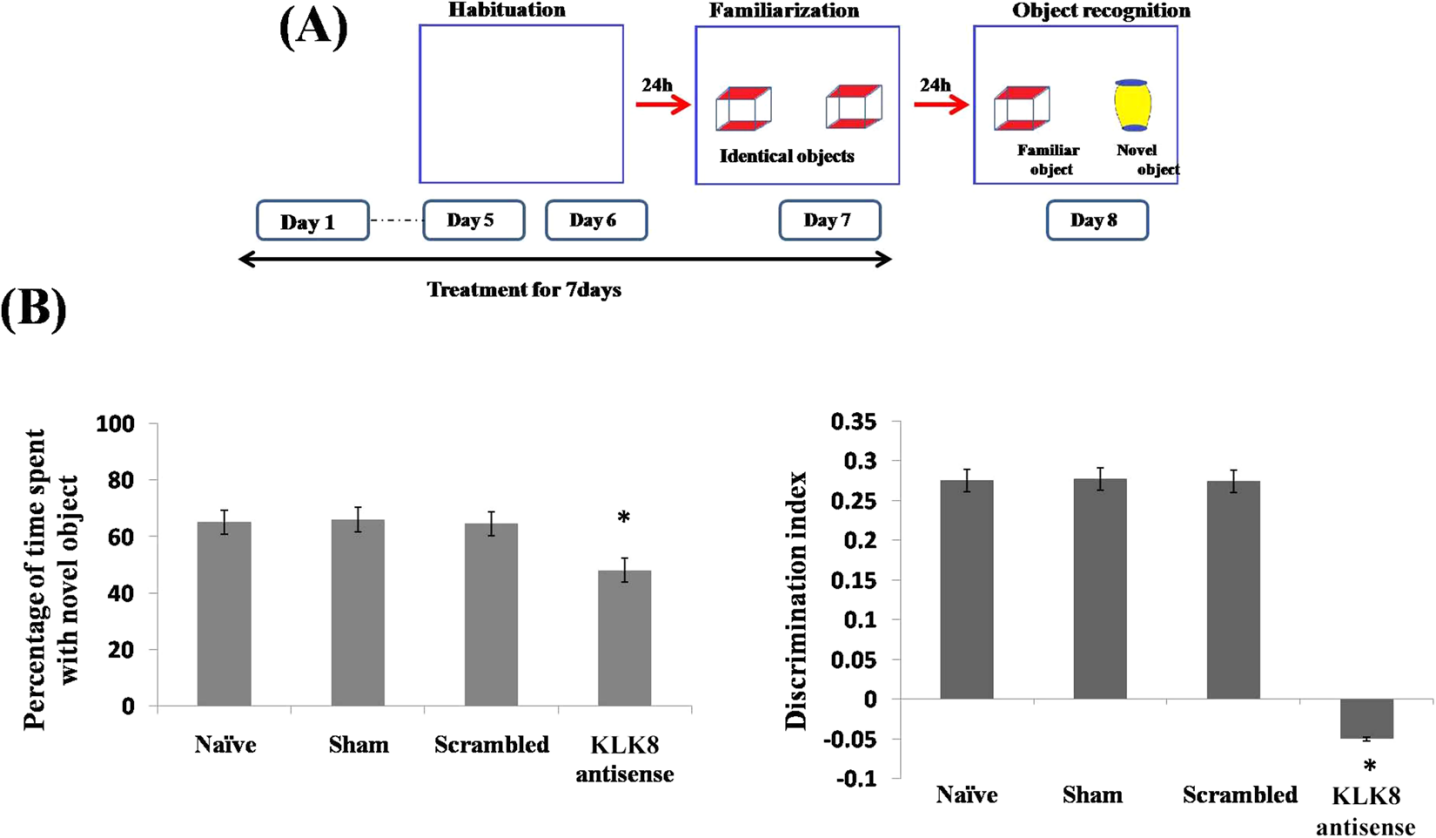
KLK8 knockdown in mouse brain impaired memory consolidation. (**A**) Experimental design of novel object recognition memory test showing (**B**) percentage of time spent with novel object and discrimination index. ‘*’ Denotes significant differences as compared to sham control.

**Figure 4:**
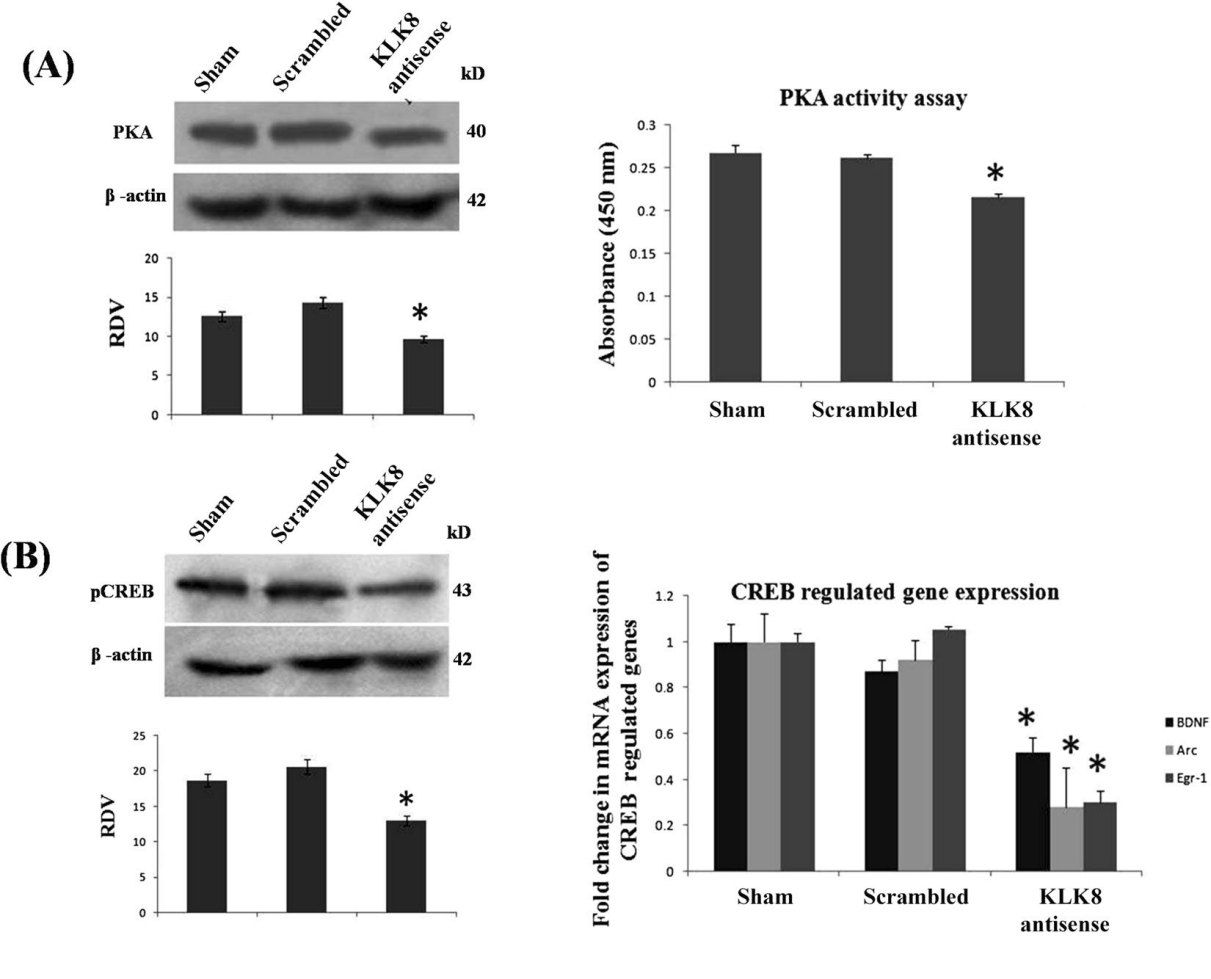
KLK8 knockdown reduced hippocampal PKA/CREB signaling. (**A**) Western blot and (**B**) enzyme activity analysis of hippocampal PKA (**C**) Western blot of hippocampal pCREB protein and (**D**) real time PCR analysis of CREB regulated genes (BDNF, Arc and Egr-1 upon KLK8 knockdown by infusion of antisense oligonucleotides. ‘*’ Denotes significant differences as compared to sham control.

Following 24 h of stereotaxic infusion of KLK8 antisense oligonucleotides, mice were familiarized with two identical objects during drug treatment for 7days and after 24 h (8th day), one object was replaced with a novel one and memory consolidation was assessed. Naive, sham and scrambled control mice spent more time with the novel object (65%) as compared to familiar one (35%), whereas mice injected with KLK8 antisense (Fig. 3) interacted for nearly equal time with both objects (48% with novel and 52% with familiar objects). KLK8 knockdown also inhibited the key PKA-CREB pathway of memory as was evident by (i) decrease in total PKA level and activity by 0.25-fold (Fig. 4A), (ii) reduction of CREB levels by 0.38-fold, and (iii) expression of CREB regulated memory permissive genes, including BDNF by 0.48-fold, Arc by 0.72-fold and Egr1 by 0.7-fold (Fig. 4B). We also analyzed pCREB normalized to total CREB, but results did not differ from those normalized to β-actin (data not shown).

## Discussion

In this study, we carefully measured mechanisms of KLK8 activity in memory processes. KLK8 gene knockdown *in vitro* and *in vivo* in mouse hippocampus revealed its novel function in microtubule associated dendrite growth and memory consolidation. Earlier studies reported that infusion of KLK8 inhibitors in mouse brain or gene knockout led to considerable impairment in plasticity by disrupting long term potentiation (LTP)^23^. KLK8 is essential to early processes of memory acquisition and LTP^10^. Matsumoto-Miyai *et al*.^18^ and Nakamura *et al*.^24^ reported that KLK8’s role in synaptic plasticity is possibly through cleavage of L1CAM. However, the precise KLK8 downstream signaling that confers its role in memory was not clear.

We observed that KLK8 deficiency in mouse hippocampus reduced L1CAM cleavage and MAP2c level and caused profound loss of dendrites. Poplawski *et al*.^25^ reported that L1CAM might influence neuronal morphology by interacting with cytoskeletal MAP proteins. Interestingly, we noted reduction only in MAP2c while other MAP isoforms were unaltered. In another independent study, we observed that KLK8 expression decreased in forebrain regions of old mice that strongly correlated with MAP2c level^26^. MAP2c is crucial for dendrite growth of developing neurons, while other heavier isoforms stabilize and maintain mature neurons^27^.

KLK8 protein levels were elevated in hippocampus of double-transgenic mice that expressed the Swedish and Indiana APP familial AD mutations under the hamster prion promoter and in human AD patient samples^28^. Likewise, suppression of KLK8 improved memory functions and synaptic plasticity in test animals, accompanied with reduced cleavage of EPHB2. This may at first blush appear contradictory, but it must be noted that they found excess KLK8 to correlate to axonal deficiency, while we found deficient KLK8 to correlate to dendritic deficiency. Herring, *et al*.^28^, also reported in passing that their control (non-transgenic) animals suffered what they term “mild adverse reactions” to KLK8 inhibition and hypothesize that KLK8’s effect on memory function may depend on both a lower and upper bound. In short, it would be possible to be pathologically deficient in KLK8, which our work suggests, or to have a pathological excess of KLK8. This is not unlike our earlier discovery that autism spectrum disorder, may be accompanied by deficiency in the AD-associated amyloid-β peptide (Aβ), in contrast to the excess associated with AD^29^. Notably, Herring’s group reported KLK8 from transgenic mice engineered to produce excess Aβ and from early-stage AD patients. Since their mouse system was not engineered to overproduce KLK8, it is reasonable to conclude that the KLK8 elevation they observed was a result of overproduction of Aβ and/or APP, and patient data might not reflect prodromal AD staging. KLK8’s participation in neurodegeneration typical of AD may rely both upon insufficiency before AD followed by excess once AD has appeared.

Dendrite growth is a prerequisite for establishment of functional neural networks and memory consolidation^30^. We observed that KLK8 knockdown impaired object recognition memory consolidation in mice by inactivating the key PKA/CREB signaling^31^. It is worth repeating that MAP2c has a binding domain for PKA and dendrite initiation and growth was enhanced by anchorage of PKA to this domain^13,14^. Also, MAP2 deficiency reduced dendritic and total PKA. As we have already noted, activation of PKA pathways induces CREB phosphorylation^15–17^. Therefore, we postulate that KLK8 secretion in synaptic cleft cleaves L1CAM, whose fragments might directly translocate to the nucleus or activate intracellular signaling cascades inducing expression of MAP2c and dendrite growth. This KLK8-dependent dendrite growth influences the PKA/CREB signaling and eventually memory consolidation.

This work is significant as it unravels a potential role for a secretory protease, KLK8, as an important regulator of memory markers and a probable therapeutic target of memory disorders. Neural protease function is not confined only to degradation of extracellular matrix but also evokes intracellular signaling cascades crucial for neural architecture and memory. Furthermore, we have elucidated KLK8 function that assumes greater importance in light of discoveries that KLK8 gene single-nucleotide polymorphisms (SNPs) associate with specific AD pathology in human patients, specifically brain and CSF Aβ and total tau protein^6^. Such a direct demonstration of KLK8 variation contributing to specific AD phenotypes would suggest that, among its many features, AD may also be seen as a disorder of neurological proteases. KLK8 would join β-secretase, and γ-secretase as a dysfunctional AD-associated protease. Downstream effects of protease dysfunction can be much larger than specific differences in their activities or expression, and they are subject to environmental perturbation^32^. The present work, being animal-centric, needs to be explored in human AD *post mortem* brain specimens for establishing translational value of this particular protease.

In general, AD research has focused on direct regulation and processing of APP. KLK8 may represent a hitherto unconsidered variable. It is also noteworthy that disrupting KLK8 signaling also disrupted excitation-inhibition (E/I) balance in the hippocampus. Disrupted E/I balance is also part of the pathogenesis of autism. We have elsewhere demonstrated several connections between APP processing and autism^29^. The memory and E/I balance functions of KLK8 could be part of a large network that interacts with neurotrophism/neural pruning provided by APP and its products, and disruption of one could lead to a cascade that disrupts the other, contributing to AD and other disorders.

Medicinal materials currently exist that may facilitate taking advantage of KLK8 activity. We have reported elsewhere that an extract of *Withania somnifera* reverses memory effects of scopolamine in mice^33^ and that this effect operates through upregulation of KLK8’s activity on the M1 muscarinic acetylcholine receptor^34^. The M1 receptor is currently under investigation as a drug target for AD^35^. Our elucidation of the enzymatic activity of KLK8 on the molecules of memory formation will aid this research.

## Methods

### Animals

All experimental procedures and protocols involving live animals were approved by the central animal ethical committee of Banaras Hindu University and followed appropriate guidelines for live animal use in research. Male Swiss albino strain mice (8 ± 1 weeks) from an inbred colony were used for the study. Animal handling and experiments were conducted in accordance with the guidelines of the Institutional and Central Animal Ethical Committees, Banaras Hindu University, Varanasi, India.

### Primary culture of mouse hippocampal neurons

Hippocampal neurons were prepared from 0-day old neonatal mice. Briefly, pups were decapitated; hippocampi were dissected out, minced, trypsin digested (0.25% trypsin, Invitrogen) and single cell suspension was prepared by vigorous trituration. The pellet was resuspended in complete neurobasal medium containing 2% B27 supplement and 0.5 mM GlutaMAX (Invitrogen). Cells were seeded at a density of 2.5 × 10^5^ cells/ml of complete medium in poly-l lysine coated culture plates and kept at 37 °C and 5% CO_2_ in a humidified incubator.

### KLK8 knockdown in cell culture by siRNA

Hippocampal neurons were transfected with 50 nM of KLK8 specific siRNA (siRNA ID-s113855, Catalog#4930771, Thermo Fisher Scientific) using Lipofectamine® RNAiMAX (Thermo) transfection reagent according to the manufacturer’s protocol. After 48 h of transfection, cells were harvested for western blotting and immunocytochemistry.

### Stereotaxic injection of KLK8 antisense into mice

Mice were anesthetized with 50 mg/kg BW sodium pentobarbital i.p. and positioned on a stereotaxic frame. KLK8 specific antisense phosphoorothioate oligonucleotides (5′-GGATTGCACAGGGTG-3′ corresponding to 500–514 bp of the KLK8 gene; Eurofins Genomics India Pvt Ltd) were stereotaxically administered (1.33 nmol/μl saline at the rate of 0.5 μl per min and injection volume of 10 μl) into the right lateral ventricle (coordinates-0.3 mm posterior and 1.0 mm lateral to the right from the bregma and 3 mm deep) of mouse brain. Mice infused with 0.9% saline and scrambled oligonucleotides (5′-CACCCTGTGCAATCC-3′, Eurofins Genomics India Pvt Ltd) served as controls. Initially animals received infusions in both sides but no effect of laterality was observed. Mice were allowed to recover for 24 h post-surgery and used for experimental purpose.

### Rapid Golgi staining

Mice were deeply anesthetized by pentobarbital (50 mg/kg BW, i.p., Sigma Aldrich) and decapitated. The brain was exposed along midline of the skull and rapid Golgi fixative was poured immediately. The brain was dissected out and immersed in fixative in dark for 5 days; rinsed several times on 5^th^ day and incubated in 0.75% AgNO_3_ solution in darkness for 48 h. Finally, 120 µm thick transverse sections were cut by a vibratome, cleared in xylene, mounted on slide and observed under a bright field microscope.

### Western blotting

Mice were decapitated as above, and hippocampal lysates were prepared by homogenizing the tissues in a buffer containing 20 mM Tris–HCl pH 7.4, 1 mM EDTA, 150 mM NaCl, and 1 mM protease inhibitor cocktail (Sigma-Aldrich) at 4 °C. Forty μg of lysates were used for western blotting using conventional methods. The primary antibodies were anti-L1CD, rabbit polyclonal (Dr. Vance Lemmon, University of Miami, USA); anti-MAP2 mouse monoclonal (M9942, Sigma-Aldrich); anti-pCREB rabbit polyclonal (Prof. Marc Montminy, Salk Institute for Biological Studies); anti-PKA (NT) (ADI-KAP-PK001, Enzo Life Sciences Inc); Monoclonal mouse anti-β-Actin (peroxidase-conjugated) (A3854, Sigma Aldrich); anti-KLK8 (M-51) rabbit polyclonal (Santa Cruz Biotechnology, Inc sc-292341). The appropriate secondary antibodies were used at adequate dilutions. MAP2c was distinguished from other MAP2 forms by molecular weight (70 kDa for MAP2c vs. 280 kDa for MAP2a and MAP2b).

### RT-PCR

RNA isolated from hippocampi of mice of different experimental groups was first reverse transcribed into cDNA using reverse transcriptase. (200 U of M-MuLv reverse transcriptase, New England Biolabs, USA) Thereafter qPCR amplification was performed using cDNA template, SYBR green master mix and gene specific primers (KLK8 FP-5′GGGTGATCATAGCCTCCAGA3′ KLK8 RP-5′TTCACTTCCGCACAGTTGAG3′; BDNF FP-5′TGCCAGAGCCCCAGGTGTGA3′ BDNF RP-5′CTGCCCTGGGCCCATTCACG3′; Arc FP-5′TATTCAGGCTGGGTCCTGTC3′, Arc RP-5′TGGAGCAGCTTATCCAGAGG3′; Egr1 FP-5′AGCGAACAACCCTATGAGCA3′, Egr1 RP-5′TCGTTTGCTGGGATAACTC3′). β-actin (FP-′GTCGTACCACAGGCATTGTG3′ RP-5′CTCTCAGCTGTGGTGGTGAA-3′) was used as an endogenous control.

### Immunocytochemistry

Cells grown on glass coverslips were washed with 1xPBS; and fixed with pre-chilled methanol: acetone (1:1 v/v) for 5–10 min at room temperature. Fixed cells were permeabilized with 0.32% Triton X-100 in PBS for 15 min, and blocked with 5% goat serum in 1xPBS for 1 h. Cells were incubated with anti-MAP2 antibody (M9942, Sigma-Aldrich) at 4 °C for 24 h, washed thrice with 0.1% Triton X-100 in 1xPBS (PBST) for 5 min each and incubated with FITC conjugated goat anti mouse secondary antibody (Sigma-Aldrich). After three washings with PBST for 5 min each, coverslips were mounted on glass slides using DAPI mounting medium. Cells were visualized by Leica inverted fluorescence microscope (Leica DFC 450 C).

### Protein kinase A assay

Protein Kinase A activity was assessed using PKA activity kit (Enzo Life Sciences Inc) in compliance with the manufacturer’s instruction. Briefly, the hippocampus was dissected out and homogenized in tissue lysis buffer (glycerolphosphate-β 20 mM; MOPS 50 mM; sodium fluoride 50 mM; sodium orthovanadate (CAS 13721-39-6) 1 mM; EGTA 5 mM; EDTA 2 mM; NP40 1% w/v; dithiothreitol (DTT) 1 mM; benzamidine 1 mM; phenylmethane- sulphonylfluoride (PMSF) 1 mM; and leupeptin and aprotinin protease inhibitor mix 10 µg/ml). After centrifugation at 10,000 ×  g, the supernatant was transferred to a microfuge tube and protein concentration was determined by BCA. For PKA assay, the kinase assay dilution buffer, purified active PKA and diluted samples were added to the appropriate wells of substrate microtiter plate. The reaction was initiated by adding diluted ATP to each well except the blank and incubated at 30 °C for 90 min with gentle shaking. The reaction was stopped by emptying contents of each well. Further, phospho-specific substrate antibody was added to each well except the blank and incubated at room temperature for 60 min with gentle shaking. After washing four times in 1X wash buffer, anti-rabbit IgG, HRP conjugated was added to each well except the blank and incubated at room temperature for 30 min with gentle shaking. The liquid was aspirated from all the wells and washed four times with 1X wash buffer. Thereafter, TMB solution was added to each well and incubated for 10 min. The reaction was stopped by adding stop solution 2 (HCl 1N) and measured the absorbance at wavelength 450 nm.

### Data processing and analysis

For *in vivo* studies, each experiment was repeated three times (n = 9 mice/group), and for *in vitro* studies, treatments were performed in three independent culture dishes and the experiment was repeated three times. To collect the data, signal intensity was measured by spot densitometry tool of AlphaEaseFC software (Alpha Innotech Corp, USA). For western blotting, the signal intensity (Integrated Density Value, IDV) of KLK8, L1CAM, MAP2, PKA and pCREB bands were normalized against signal intensity of internal control β-actin and plotted as relative density value (RDV). No gels were grouped for presentation. Full-length images of all blots are in Supplemental materials (S2–S4). No blots were overexposed. In order to analyze qRT-PCR data, the ΔΔCt value was used to calculate relative fold change in KLK8 mRNA expression and plotted as histograms. Dendrite growth was analyzed by measuring the average length and number of dendrites using Leica LASV4.2 software. Microscopic images from random fields were captured and length and number of dendrites were quantified and expressed as total length and total number of dendrites per treatment group. Average length and number of dendrites was obtained by dividing total length or total dendrites by the number of cells within a given field. For pairwise comparison, student’s t-test was used using SigmaPlot, version 2.0, Jandel Scientific software. Values were reported as mean ± SEM and p values < 0.05 were considered as significant.

## Electronic supplementary material

Supplementary Information
