# Supplementary material for: A serine protease KLK8 emerges as a regulator of regulators in memory: Microtubule protein dependent neuronal morphology and PKA-CREB signaling
Source: Sci Rep. 2018 Jul 2;8:9928. doi: 10.1038/s41598-018-27640-6 (PMC6028475; doi:10.1038/s41598-018-27640-6)
Supplement: Supplementary file 1 — Supplementary Information [file 41598_2018_27640_MOESM1_ESM.pdf]

A serine protease KLK8 emerges as regulator of regulators in memory: Microtubule protein dependent neuronal morphology and PKA-CREB signaling

Arpita Konar, Ashish Kumar, Bryan Maloney, Debomoy K. Lahiri, and Mahendra K. Thakur

Supplementary Material

S1. DAPI staining assessment of nuclear morphology and light microscopy of cultures.

S2-S4. Full-length images of blots presented in main paper.

S1

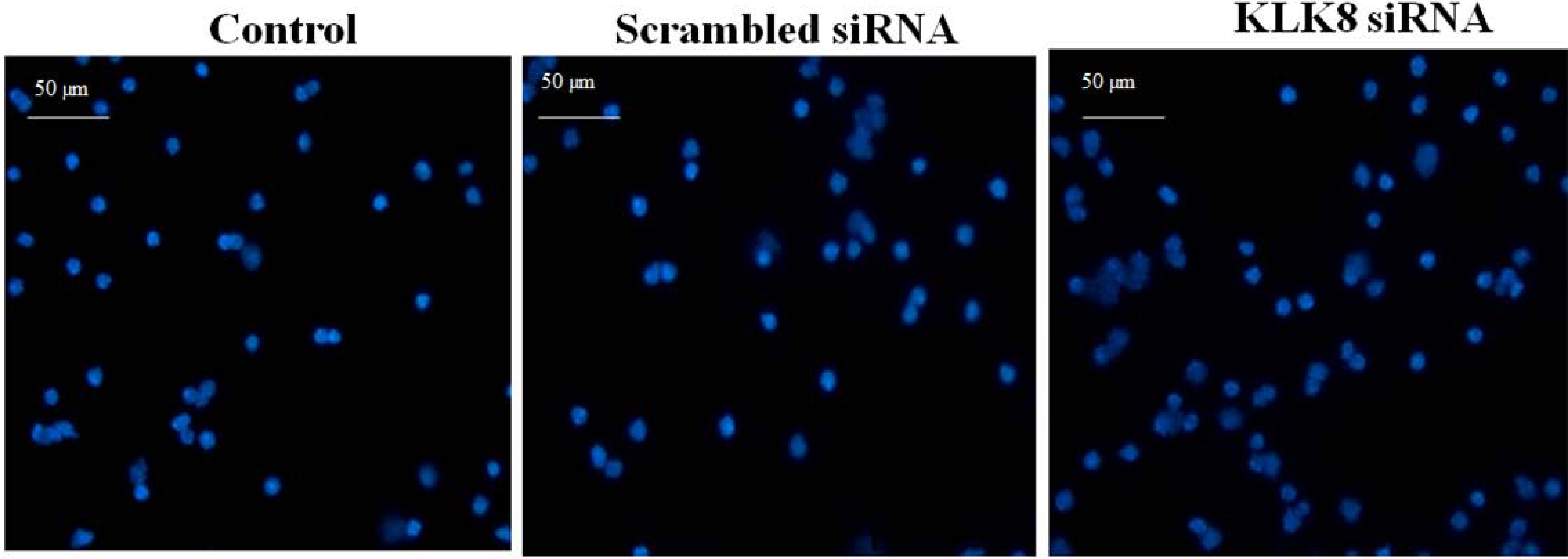

S2

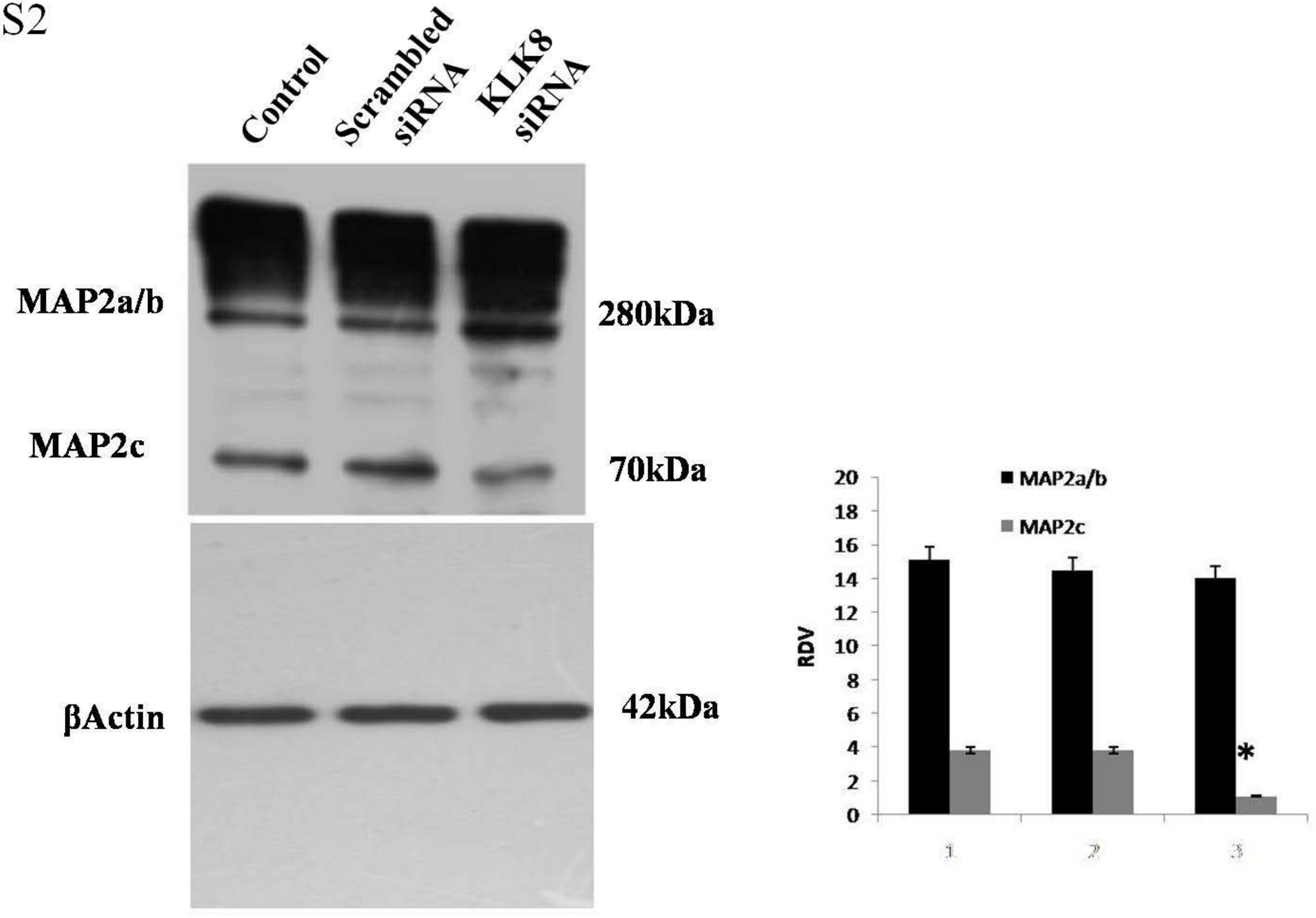

Control
Scrambled siRNA
KLK8 siRNA
MAP2a/b
MAP2c
βActin
280kDa
70kDa
42kDa
MAP2a/b
MAP2c
RDV
0
2
4
6
8
10
12
14
16
18
20
1
2
3
*

S3

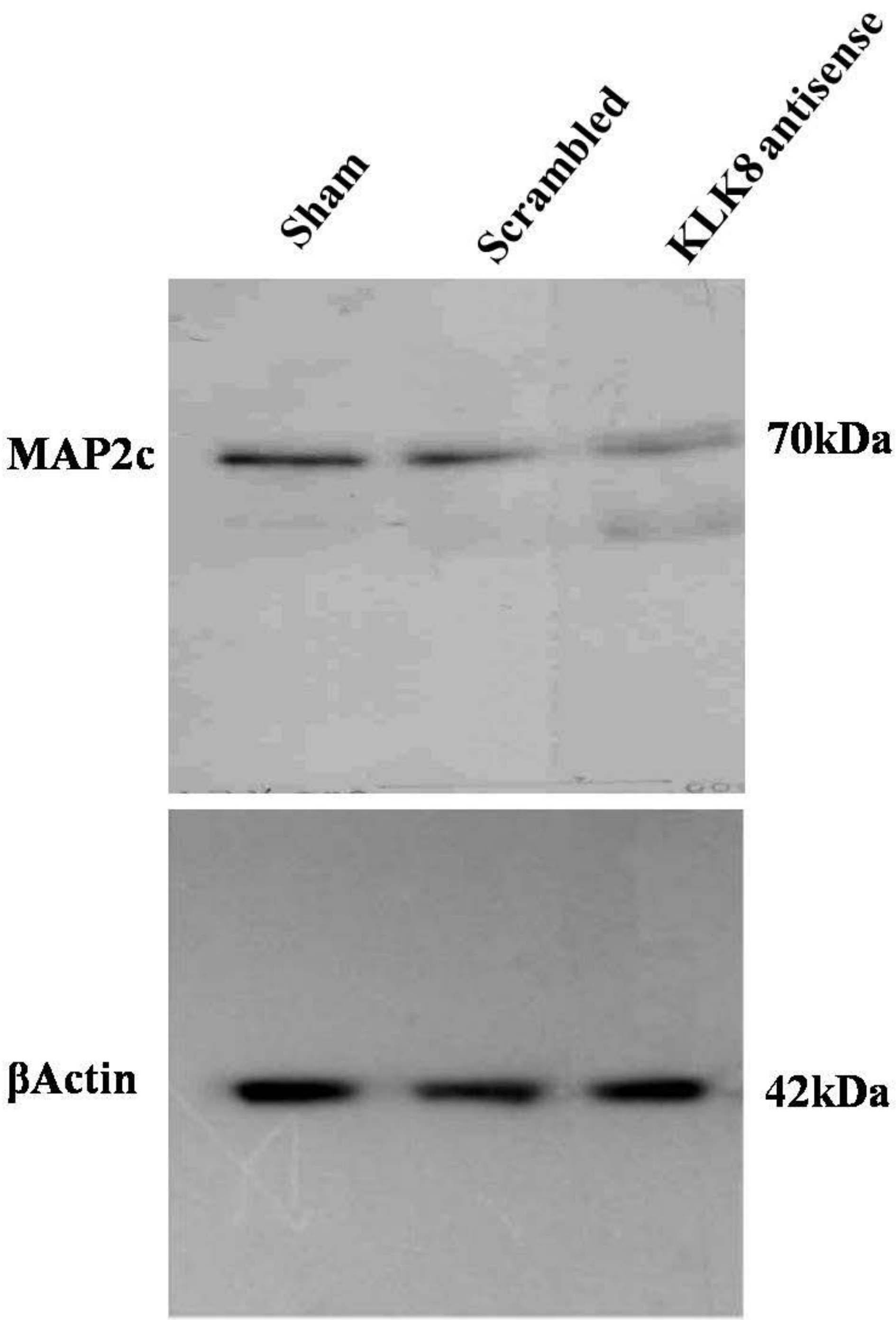

S4

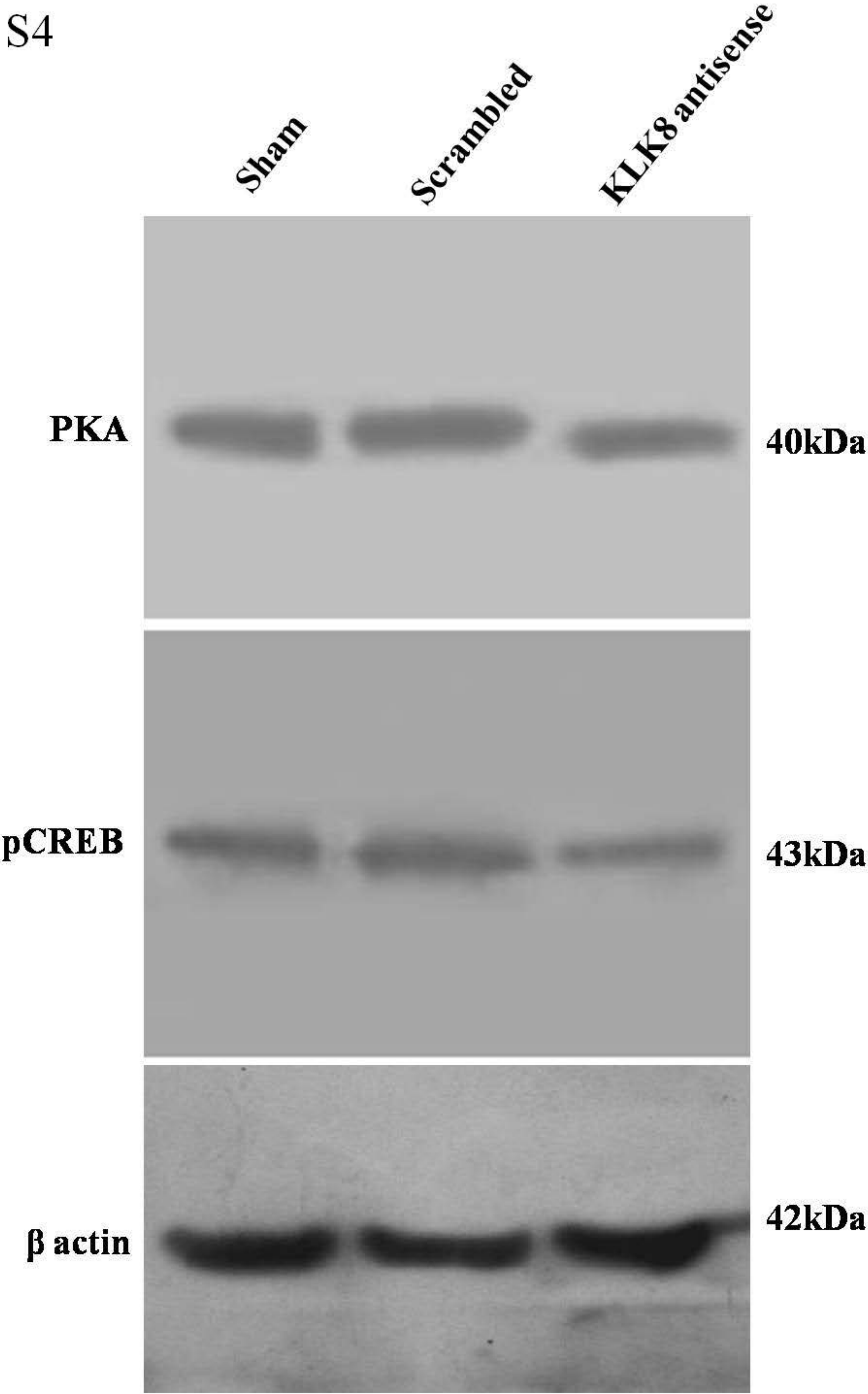
Sham
Scrambled
KLK8 antisense
PKA
40kDa
pCREB
43kDa
β actin
42kDa
